# Dr. Franklin R. Pitner

**Published:** 1912-12

**Authors:** 


Dr. Franklin R. Pitner died at his home in Clay City, Ill., Sept. 29, lacking 12 days of being 99 years old. He graduated at the old Transylvania Medical College. Except for two years spent in California soon after the discovery of gold, his life was spent in Illinois, and he was engaged in active practice till he was 90.

